# Group medicare wellness visits: A pilot exploration of an approach to wellness and preventive medicine for older adults

**DOI:** 10.1016/j.pmedr.2023.102514

**Published:** 2023-11-10

**Authors:** Charis Wiltshire, Katarzyna Budzynska, Pooja Kulkarni, Nike Shoyinka, Denise White Perkins

**Affiliations:** a Department of Family Medicine and Public Health Sciences, Wayne State University School of Medicine, 939 Woodward Avenue, Detroit, MI 48201, United States; b Department of Family Medicine, Henry Ford Hospital, 3370 E Jefferson Ave, Detroit, MI 48207, United States

**Keywords:** Healthy aging, Medicare wellness, Patient satisfaction, Shared medical appointment, Health maintenance gap, Chronic disease management

## Abstract

**Introduction:**

The Medicare annual wellness visit was designed to address health risks and encourage evidence-based preventive care in aging. However, it can be challenging for providers to dedicate time for comprehensive attention to wellness during these visits. Our project implements a group setting for Medicare wellness visits (GMWV) as an efficient method for delivering high value preventive care.

**Methods:**

Three hundred patients from two primary care ambulatory clinics in Detroit, MI in need of their annual Medicare visit were invited to participate in the pilot GMWV. Fifty-eight patients agreed and completed their GMWV. The visit included collection of vitals, vision screening, and risk assessment during check-in, followed by educational wellness presentations led by an interdisciplinary team of six healthcare professionals. Patients completed a post visit-satisfaction survey and researchers calculated rates of completion of health maintenance gaps (HMG), i.e. immunizations and cancer screenings, among participants.

**Results:**

The average age of participants (N female = 48) was 74 years old. Thirty-four participants had more than one HMG at baseline. On average, 8 % of immunization gaps and 12 % of screening gaps were completed at or within one-year post GMWV. Participant feedback reported that 82 % of patients felt that they learned something new from the presentation and 81 % of patients felt satisfied with the amount of time they spent with their physician.

**Discussion:**

GMWV is a feasible approach to promoting wellness and healthy aging that patients find satisfying although, additional study is needed to compare the effectiveness of this model to standard care.

## Highlights

• The Medicare annual wellness visit was implemented to address health risks yet providers still struggle to find dedicated time for comprehensive attention to wellness during these visits.
• Our project investigates the feasibility of Medicare wellness visits performed in the group setting.
• Additional study is needed to compare the effectiveness of this model to standard care.

## 1 Introduction

The annual Medicare wellness visit (MWV) was introduced in 2011 as a part of the Affordable Care Act and Patient Protection initiatives to facilitate preventive services to the older adult population (Colburn and Nothelle, 2018). Given that 16.9 % (U.S. Census Bureau, 2022) of the United States population are over 65 years old, and many of these individuals are living with at least one chronic medical condition, demands for chronic disease management and time dedicated to comprehensive attention to wellness are significant (Ahmed, 2016). A study from 2018 found that MWV utilization increased from 8.1 % in 2011 to 23.0 % in 2016, but that utilization was significantly lower among racial and ethnic minorities (Lind et al., 2019). These studies suggest that overall utilization of the MWV is increasing but persons from minoritized communities are still falling behind.

To increase access to MWV among our older adult primarily Black patients in Detroit, thereby increasing our ability to address health maintenance gaps (HMG), our team adapted these visits to a shared medical appointment group visit format (Noffsinger, 1999). Group visits have been shown to lead to positive health outcomes through improved patient experience (Cunningham et al., 2021), specifically in patients with chronic pain (Lestoquoy et al., 2017), attempting weight loss (Axten et al., 2017) as well as for patients who are older (Cherniack, 2014) and needing advanced care planning (Lum et al., 2016, Lum et al., 2017). Group visits effectiveness could be due to increased patient satisfaction (Egger et al., 2015, Heyworth et al., 2014) and easing primary care provider burden (Egger et al., 2015, Stults et al., 2016). A preliminary report of a group MWV (GMWV) piloted in 2013 explored benefits and challenges from the provider’s perspective of the group visit format and found high ratings of patient satisfaction and comfort (Kainkaryam, 2013).

Understanding the patient characteristics and program features that may promote or inhibit completion of key health maintenance activities in older adults, such as immunizations and routine screenings, should help institutions create effective wellness initiatives to help this patient population fill important gaps in health maintenance. Therefore, our goal was to give further evidence to the feasibility of a (GMWV) program for older adult patients in the ambulatory primary care centers of our large integrated health system located in Detroit, Michigan. Here we describe our intervention, the clinical and demographic characteristics of the patient participants, and discuss the utility of using such an approach for addressing HMGs.

## 2 Materials and methods

### 2.1 Patients and inclusion criteria

Participants were identified by five primary care providers available at the time of the sessions across two clinics in Detroit, MI whose patients were enrolled in Medicare and due for an annual MWV. Inclusion criteria for this pilot study included: current enrollment in Medicare insurance; no MWV within the last year; sufficient hearing and vision to participate in a group setting per medical records review; and no diagnosis of severe dementia. All eligible patients were invited to sessions led by their primary care physician by mail approximately 3 weeks prior to the GMWV, followed by a reminder telephone call 10 and 2 days prior to the visit. Sessions took place between December 2017 and September 2019.

### 2.2 Program implementation

Upon arrival, a medical assistant conducted a check-in with each patient, consisting of vital signs and vision screening, and patients were left to complete the Medicare health risk assessment questionnaire individually (45 min allotted). A nurse educator who served as the central facilitator began the visits in a large conference room by briefly orienting patients to the group visit format. This was followed by a series of six interactive educational presentations led by the physician, physical therapist, nurse educator, pharmacist, dietician, and social worker (10 min each; total 60 min). Topics were consistent between sites though led by different presenters and ranged from screenings and immunizations, fall prevention, medication safety/adherence, community resources for older adults, and a nutrition demonstration half of which offered food to participants while water was available at all sessions. The presentation closed with a question-and-answer portion (15 min). Patients were encouraged to interact with speakers and with other patients as each waited for a one-on-one health maintenance review with their physician in a private examination room. During these brief visits (5–10 min), the physician ordered appropriate screening tests and referrals which required a separate appointment, and immunizations that could be completed at the time of the visit. Upon discharge, patients were asked to complete an anonymous survey that queried satisfaction with participating in the GMWV and were given educational materials.

### 2.3 Data collection and analysis

All research activities were approved by the Henry Ford Health System Institutional Review Board (#13526-29). Demographic (age, sex, body mass index, race, type of insurance, marital status, and smoking status) and clinical information (to calculate the Charlson Comorbidity Index) were abstracted from patients’ electronic health records and recorded in REDCap (Harris et al., 2019). Because most of the patients self-identified as Black/African American per the electronic medical record, we stratified patients into only two racial categories: Black/African American and “Other.”

Each patient’s HMG were defined into 2 categories: immunizations (pneumonia, tetanus/Tdap, and shingles) and screenings (mammogram, bone density, colonoscopy, and cervical cancer screening). HMGs were defined as being past due according to the U.S. Preventive Services Task Force guidelines (U.S. Preventive Services Task Force, 2023). Completion of HMGs was assessed at the GMWV (index visit) and within one year following the index visit, allowing ample time for completion of screenings that require more preparation and scheduling such as colonoscopies and mammograms.

The Medicare Health Risk Assessment (MHRA) was administered at the beginning of the visit while patients waited after check-in. The 31-item scale assesses risk related to performing daily activities, emotional problems, and behavioral risk factors. The satisfaction survey asked participants to rate various aspects of the session and each of the specific talks. Questions about how much time the patients felt that they had with the various healthcare providers and whether they felt that the presenters were knowledgeable were included in each section of the survey. The anonymous and de-identified surveys were collected on paper and later transcribed to be stored electronically; results were subsequently summarized to explore general trends. The survey was divided into subscales to explore specific aspects of the patient’s experience. Copies of the MHRA and satisfaction survey as well as coding of the subscales can be found in the Supplement.

For analysis purposes, patients were stratified into two groups: those who had 1 or no HMGs at the index visit, and those who had more than 1 HMG at the index visit. Groups were compared to assess the association of demographic and clinical factors associated with number of HMGs (low versus high). Fisher’s exact test was used to assess differences in categorical variables and one-way analysis of variance was used to assess differences in continuous variables.

## 3 Results

Over 300 charts were reviewed to find eligible patients and roughly 30 invitation letters were sent out for each of the 10 visits, resulting in a total of 58 patients aged 74 years (standard deviation 4.88 years) who participated in the GMWV (48 women and 10 men). Each visit had an average of 5.7 (minimum 3, maximum 9) patients in attendance. At the index visit, there were 34 patients (59 %) who had >1 HMG and 24 patients (41 %) who had ≤1 HMG. We observed no significant differences in demographics or risk assessments between patients who had >1 HMG and those who ≤1 HMG (Table 1).

**Table 1:** Descriptive statistics of patient demographic characteristics by HMG status from two primary care clinics in Henry Ford Hospital System, Detroit, MI: 2017–2019.

Characteristic (N = 58)		HMG ≤ 1 n = 24	HMG > 1 n = 34	*p* value
Age*,* years, mean (SD)		73.0 (9.0)	75.7 (7.0)	0.202
BMI*,* kg/m^2^, mean (SD)		31.4 (7.2)	33.2 (10.2)	0.453
CCI, mean (SD)		4.88 (1.9)	5 (10.2)	0.822
Medicare Health Risk Assessment Score, mean (SD)	Total	18.8 (9.6)	22.5 (11.3)	0.195
	Self-assessment of health	9.0 (4.4)	11 (4.8)	0.11
	Psychosocial risks	4.6 (4.2)	5.2 (4.5)	0.62
	Behavioral risks	5.1 (3.0)	6.23 (4.5)	0.283
Sex, % of total	Female	18 (75)	30 (88)	0.291
	Male	6 (25)	4 (12)	
Race, % of total	Black/African American	20 (83)	30 (88)	0.706
	Other	4 (17)	4 (12)	
Patient insurance, % of total	Private	0 (0)	0 (0)	0.712
	Medicare	2 (50)	2 (50)	
	Medicaid	1 (100)	0 (0)	
	Medicare & Medicaid	2 (67)	1 (33)	
	Private insurance & Medicare	18 (38)	29 (62)	
	Private insurance & Medicare & Medicaid	1 (33)	2 (67)	
Marital status, % of total	Not married/living as single	6 (25)	5 (15)	0.098
	Married/living as single	11 (46)	11 (32)	
	Divorced/separated	2 (8)	12 (35)	
	Widowed/widower	4 (17)	6 (18)	
	Unknown	1 (4)	0 (0)	
Smoking status, % of total	Never	10 (42)	13 (38)	0.625
	Past	11 (46)	19 (56)	
	Current	3 (13)	2 (6)	

BMI, body mass index; CCI, Charlson comorbidity index; HMG, health maintenance gap; SD, standard deviation.

Health maintenance gaps are defined as patient was overdue for one or more of the following preventive health screenings: mammogram, colonoscopy, or cervical cancer screening.

Medicare Health Risk Assessment (MHRA) Self-Assessment of Health Status subscale included questions like: “How would you rate your health in general?”.

MHRA Psychosocial Risks subscale included questions like: “Have you been unable to relax?”.

MHRA Behavioral Risks subscale included questions like: “Do you use tobacco products?”.

Full documentation of the MHRA and subscale creation can be found in Supplement.

HMG completion rates are shown in Table 2. At the index visit, all 58 patients were eligible to receive all 3 immunizations, and while most patients had already had the pneumonia (n = 48; 83 %) and tetanus/Tdap (n = 45; 78 %) vaccinations, only 4 (7 %) patients had received the shingles vaccine. Of the patients who had not completed these immunizations before the index visit, 6 of 10 (60 %) received the pneumonia vaccine, 2 of 13 (15 %) received the tetanus/Tdap vaccine, and only 5 of 54 (9 %) received the shingles vaccine at or within 1 year of the index visit.

**Table 2:** Distribution summary of completion of health maintenance gaps among patients from two primary care clinics in Henry Ford Hospital System, Detroit, MI: 2017–2019.

Category	Health Maintenance Gap	Patients Eligible for ServiceN (%)	Service Complete Before Index Visitn (% of eligible)	Service Not Complete Before Index Visitn (% of eligible)	Completed Service at or within 1 Year of Index Visitn (%)*
*Immunizations*	Pneumonia	58 (100)	48 (83)	10 (17)	6/10 (60)
	Tetanus/Tdap	58 (100)	45 (78)	13 (22)	2/13 (15)
	Shingles	58 (100)	4 (7)	54 (93)	5/54 (9)

*Screenings*	Mammogram	58 (100)	31 (53)	27 (47)	2/27 (7)
	Bone density	27 (47)	13 (48)	14 (52)	5/14 (36)
	Colonoscopy	52 (90)	34 (65)	18 (35)	1/18 (6)
	Cervical cancer	4 (7)	2 (50)	2 (50)	1/2 (50)

Note that not all patients were eligible or were recommended for certain screenings (i.e., based on age or sex).

* Denominators in the last column represent the number of patients who had not completed the service prior to the index visit.

Approximately half of the patients who were eligible for the four types of screenings had completed them before the index visit. Colonoscopy screening had the highest rate of completion before the index visit (34/52; 65 %), while bone density screening was the lowest (13/27; 48 %). Of those eligible for screenings with gaps remaining, 5 of 14 (36 %) completed bone density screening, 1 of 2 (50 %) completed cervical cancer screening, only 2 of 27 (7 %) completed a mammogram, and only 1 of 18 (6 %) completed a colonoscopy within one year of the index visit (Table 2).

A total of 37 (63.8 %) patients completed satisfaction surveys. Overall, 78.4 % of patients strongly agreed that they enjoyed the session. The physician wellness and the fall prevention presentations were the highest rated, both at 86.4 % satisfaction. The knowledge of the presenters was the highest rated subscale at 83.0 %, with patient learning impact and patient physician time tied for second at 81.0 % satisfaction. Specific areas for improvement were also identified, as only 46.0 % of patients strongly agreed that the discussion with the pharmacist was helpful.

## 4 Discussion

Our initial pilot program suggests that a multidisciplinary group MWV model may be a feasible approach for promoting wellness and health maintenance tailored to the needs of older adults. While most patients were satisfied with the program overall, results showed that information about general wellness and fall prevention was the most appreciated, and discussion with pharmacists was seen as the least helpful. Our findings also suggest that certain HMGs may be more difficult to address than others.

Previous studies have shown an increase in preventive screenings for those who participated in MWV (Camacho et al., 2017, Chung et al., 2018). We looked at 9 different preventive services and noted variations in service completion both before and after the program. For example, we saw higher completion rates in pneumonia vaccines and bone density screening, yet we note that few patients received the shingles vaccine before or after being in the program, which could be due to cost, mistrust or low perceived severity or susceptibility. Additionally, while most eligible patients (65 %) already had a colonoscopy before the program, only 1 of the 18 patients who were due for this procedure completed it within one year of program participation. Our findings contribute to existing research suggesting that older patients may have differing views on the various health maintenance services recommended by physicians, and a more thorough effort explaining the benefits of certain services, especially the shingles vaccine, may be needed (Brown Nicholls et al., 2021).

Overall, patient feedback indicated that participants were satisfied with the detailed yet digestible information, highlighting that they were engaged, willing, and happy to learn. The group format and the time reserved for inter-group interaction allowed older adult patients to interact with their peers, share perspectives, and exchange valuable information regarding wellness practices and community resources.

We acknowledge several opportunities for improvement in implementation, including a more streamlined checkout process and an increased transparency regarding the program agenda so that patients will know what to expect during the visit. This could include telephone calls prior to the visit to inquire about their specific wellness questions so that we might tailor the educational content to their needs.

Our preliminary analysis described the characteristics of patients who participated in a GMWV program. However, we acknowledge that our study design did not allow us to make causal inferences about the impact of the GMWV on closing HMGs. Cost-benefit studies with analysis of the most efficient staffing model would also be important for determining the return on investment for a group care delivery model.

### 4.1 Conclusions

Overall, we are confident that the GMWV approach to preventive healthcare for older adults is an innovative strategy, which promises to be an efficient and effective model for addressing inequities in care. The group format not only allows patients to interact with each other to share feelings, information, and experiences, but also offers patients access to different healthcare providers who can answer a range of questions on different topics. Thus, a group approach to MWVs can save patients time and effort by addressing multiple HMGs in one event and promote a shared community approach to preventive healthcare.

### CRediT authorship contribution statement

**Charis Wiltshire:** Formal analysis, Data curation, Writing – original draft, Visualization. **Katarzyna Budzynska:** Conceptualization, Investigation, Writing – review & editing, Supervision. **Pooja Kulkarni:** Project administration, Investigation. **Nike Shoyinka:** Project administration, Investigation. **Denise White Perkins:** Conceptualization, Investigation, Writing – review & editing, Supervision.

## Declaration of Competing Interest

The authors declare that they have no known competing financial interests or personal relationships that could have appeared to influence the work reported in this paper.

## Data Availability

Data will be made available on request.
